# The role of the ADVanced Organ Support (ADVOS) system in critically ill patients with multiple organ failure

**DOI:** 10.1111/aor.14188

**Published:** 2022-02-06

**Authors:** Metesh Acharya, Rafal Berger, Aron‐Frederik Popov

**Affiliations:** ^1^ Department of Cardiac Surgery Glenfield Hospital Leicester UK; ^2^ Department of Thoracic and Cardiovascular Surgery University Hospital of Tübingen Tübingen Germany; ^3^ Department of Cardiac Surgery Helios Clinic Siegburg Siegburg Germany

**Keywords:** ADVanced Organ Support, albumin dialysis, extracorporeal organ support, multiple organ failure, multiple organ support

## Abstract

**Background:**

Multi‐organ failure characterized by acute kidney injury, liver dysfunction, and respiratory failure is a complex condition associated with high mortality, for which multiple individual support devices may be simultaneously required. This review aims to appraise the current evidence for the ADVanced Organ Support (ADVOS) system, a novel device integrating liver, lung, and kidney support with blood detoxification.

**Methods:**

We performed a literature review of the PubMed database to identify human and animal studies evaluating the ADVOS system.

**Results:**

In porcine models of acute liver injury and small clinical studies in humans, ADVOS significantly enhanced the elimination of water‐soluble and protein‐bound toxins and metabolites, including creatinine, ammonia, blood urea nitrogen, and lactate. Cardiovascular parameters (mean arterial pressure, cerebral perfusion pressure, and cardiac index) and renal function were improved. ADVOS clears carbon dioxide (CO_2_) effectively with rapid correction of pH abnormalities, achieving normalization of CO_2_, and bicarbonate levels. In patients with COVID‐19 infection, ADVOS enables rapid correction of acid–base disturbance and respiratory acidosis. ADVOS therapy reduces mortality in multi‐organ failure and has been shown to be safe with minimal adverse events.

**Conclusions:**

From the small observational studies analyzed, ADVOS demonstrates excellent detoxification of water‐soluble and protein‐bound substances. In particular, ADVOS permits the correction of metabolic and respiratory acidosis through the fluid‐based direct removal of acid and CO_2_. ADVOS is associated with significant improvements in hemodynamic and biochemical parameters, a trend toward improved survival in multi‐organ failure, and is well‐tolerated. Larger randomized trials are now necessary to further validate these encouraging results.

## Graphical abstract

The novel ADVanced Organ Support (ADVOS) system enables effective elimination of water‐soluble and proteinbound toxins and metabolites, providing kidney, liver and lung support in a single integrated device in multi‐organ failure. In both animal and human studies, ADVOS significantly reduces serum bilirubin, ammonia, creatinine, lactate and blood urea nitrogen levels, whilst correcting acid‐base disturbances through blood pH, bicarbonate and carbon dioxide normalisation. ADVOS therapy is less invasive, well‐tolerated with a minimised side effect profile, and reduces mortality in multi‐organ failure.

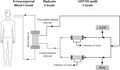

## 1 INTRODUCTION

Multiple organ failure syndrome (MOFS) remains a substantially challenging condition and the leading cause of death in intensive care settings. It is characterized by the progressive deterioration of more than one organ system evolving through a complex interplay of pathophysiological mechanisms, including oxygen delivery/consumption mismatch, release of inflammatory and vasoactive mediators, and destabilization of intestinal mucosal barrier function.^1^ Failing organs propagate the systemic inflammatory response.^2^ While our understanding of MOFS has developed in the past few decades, the associated mortality remains high, exceeding 50% when two organs fail and 75% with concurrent hepatic, respiratory, and renal failure.^3^ Treatment centers around early and aggressive resuscitation for the restoration of tissue perfusion, control of superimposed infection, timely, and definitive surgical management, and nutritional maintenance.

Multiple systems for the support of individual organs, including those for renal replacement therapy and extra‐corporeal lung and liver support, may be simultaneously indicated to sustain several failing organs,^1^ although this complicated approach often presents additional logistical difficulties in the management of highly complex and critically ill patients. However, this is not new. In the 1950s renal replacement therapy (RRT) became available,^4^ while continuous renal replacement therapy (CRRT) first appeared in the late 1970s.^5^ Although the risks and benefits of RRT have been established for decades, debate still exists regarding the optimal time to commence this therapy.^6^

During the late 1990s and the 2000s, marketing of extracorporeal liver support systems to eliminate protein‐bound toxins began. These include albumin dialysis therapies such as single‐pass albumin dialysis (SPAD) and the Molecular Adsorbent Recirculating System (MARS®), the fractionized plasma separation and adsorption system (Prometheus®), high‐volume plasma exchange (HVP), extracorporeal bioartificial cellular therapies using extracorporeal liver cell bioreactors for blood purification and more recently, hemadsorption.^7^ Initial clinical studies demonstrated multiple benefits, although the results of subsequent randomized clinical trials reduced the initial euphoria.^8^ However, a recent meta‐analysis reported a benefit in mortality in patients with acute liver failure and acute‐on‐chronic liver failure.^9^

Regarding lung support, extracorporeal membrane oxygenation (ECMO) was first performed successfully in the 1970s by Hill,^10^ leading on from previous work by Gibbon^11^ and Kolobow,^12^ among others. A “low‐flow ECMO” or extracorporeal carbon dioxide (CO_2_) removal (ECCO2R) conceived to reduce the invasiveness and risks of conventional ECMO by Gattinoni^13^ in the 1980s is still struggling to gain market acceptance, especially after the last negative randomized controlled trial.^14^

These single‐organ support devices may be implemented as and when required to support failing organ systems and are familiar to the intensive care staff accustomed to their daily use. However, the use of multiple single‐organ support devices can mandate a large extra‐corporeal volume, is cumbersome, requires additional time for their set‐up and maintenance, and is associated with significant financial implications.^7^ This being said, many authors point toward a multiple organ support therapy (MOST), which would integrate several single‐organ support systems into one device^15^, ^16^, ^17^ to limit extra‐corporeal blood volume and reduce operator effort. Such a system would ideally be able to simultaneously support the lung (for CO_2_ removal and increased oxygenation), the heart (through circulatory support), the kidney (hemodialysis), and the liver (albumin dialysis).^17^

The ADVanced Organ Support (ADVOS) system (ADVOS multi, ADVITOS GmbH, Munich, Germany), a novel albumin dialysis therapy, claims to support three of these organs (lung, liver, and kidneys), albeit without providing oxygenation. Facility for the correction of acid–base balance disturbances can additionally be provided. The purpose of this review, therefore, was to evaluate the scientific literature available regarding the ADVOS system, discussing its strengths, limitations, and future challenges.

## 2 LITERATURE SEARCH

A literature search was performed using the PubMed database for the terms “Hepa Wash,” “Advanced Organ Support,” and “ADVOS.” Peer‐reviewed publications that have been indexed in PubMed are further discussed throughout this review, as listed in Table 1. Additionally, the website of the manufacturer (https://www.advitos.com/en/mediathek/) was checked to include other relevant publications which are not indexed in PubMed, as listed in Table S2).

**TABLE 1:** List of peer‐reviewed publications with ADVOS therapy and summary of the main outcomes

Study	*N* (ADVOS/control)	Liver support	Kidney support	Lung support	Acid–base balance correction	Additional features	Adverse events	Survival	Ref
*Preclinical*									
Al‐Chalabi, 2013	6/5	NH_3_: 562 vs. 1382 μg/dl^a^, N/N: 5.54 vs. 49.82 μmol/L^a^	UO: 1850 vs. 420 ml^a^, SCr: 1.3 vs. 3.2 mg/dl^a^	N/A	N/A	CPP: 23 vs. 10 mm Hg^a^, MAP: 37 vs. 24 mm Hg^a^, CI: 4.94 vs. 3.36 ml/min/m^2a^	None attributed to Hepa Wash	83% vs. 20%^a^	^23^
Al‐Chalabi, 2017	5/5	Bilirubin, 2.3 vs. 5.5 mg/dl^a^, NH_3_: 194 vs. 681 μg/dl^a^, Lactate: 4.2 vs. 8.3 mmol/L^a^	BUN: 6 vs. 17 mg/dl^a^, SCr: 1.4 vs. 2.3 mg/dl^a^	FiO_2_: 49% vs. 82%^a^		CI: 6.7 vs. 4.9 ml/min/m^2a^, CPI: 0.83 vs. 0.41 W/m^2a^	None related to ADVOS	100% vs. 0%^a^	^25^
Perez, 2019	N/A	N/A	N/A	ECCO2R: up to 142 ml/min	Acidosis correction within 1 h	N/A	N/A	N/A	^21^
*Clinical trials and case series*									
Huber, 2017	14/0	Bilirubin: 17.7 vs. 26.0 mg/dl^b^	BUN: 31.1 vs. 49.4 mg/dl^b^, SCr: 1.6 vs. 2.2 mg/dl^b^	n.d.	n.d.	n.d.	None related to ADVOS	28‐day: 43%, Expected: <20%^d^	^26^
Fuhrmann, 2020	34/0	Bilirubin: 17% reduction, NH3: 16.4% reduction	BUN: 17.6% reduction, SCr: 7.1% reduction	PaCO_2_: 54 vs. 69 mm Hg^b^, DP: reduced in 75% of the sessions	pH (resp. acidosis): 7.40 vs. 7.21^b^, pH (met. acidosis): 7.40 vs. 7.19^b^, Acidosis correction within 6 h	MAP: 69 vs. 74 mm Hg^b^	None related to ADVOS	28‐day: 50%, 90‐day: 38%, Expected: <10%^d^	^22^
Falkensteiner, 2021	18/31	Bilirubin: 15% reduction	SCr: 18% reduction, BUN: 21% reduction	N/A	N/A	N/A	Bleeding (not specified if directly related to ADVOS)	ICU: 45 vs. 39%^a^	^28^
Kaps, 2021	26/25^c^	Bilirubin: 14.5% reduction	SCr: 11.8% reduction, BUN: 33.7% reduction	N/A	N/A	N/A	N/D	28‐day: 56 vs. 40%^c^, Expected: 56%^e^	^29^
Fuhrmann 2021	118/0	Bilirubin: 6.5 vs. 6.9 mg/dl^b^	SCr: 1.2 vs. 1.5 mg/dl^b^, BUN: 17 vs. 24 mg/dl^b^	N/A	Base excess: 1.7 vs. −3.5 mmol/L^b^, ${\mathrm{HCO}}_{3}^{-}$: 25.8 vs. 22.1 mmol/L^b^	N/A	Clotting	28‐day: 39.7%, 90‐day: 35.4%, Expected: <20%^d^	^33^
Scharf, 2021	6/33	Bilirubin: 22.8% vs. 22.5% reduction^a^	N/A	N/A	N/A	N/A	N/A	In‐Hospital: 33.3% vs. 15.2%^a^	^30^
*Case reports*									
Jarczak, 2019	1/0	N/A	N/A	N/A	N/A	Myoglobin: 40.9% (50.5% combined with hemadsorption), CK: 62.2% (80.8% combined with hemadsorption)	N/D	N/A	^31^
König, 2021	1/1	N/A	N/A	N/A	N/A	Meropenem *t*_1/2_: 2.2 vs. 3 h^a^, Meropenem CL_T_: 8.6 vs. 4.6 L/h	N/D	N/A	^32^
*COVID‐19*									
Huber, 2020	1/0	N/A	N/A	ECCO2R: 48 ml/min	Blood pH: 7.29 vs. 7.19^b^	NA: 0.04 μg/kg/h vs. 0.35 μg/kg/h^b^	N/D	The patient died	^34^
Allescher 2021	9	N/A	SCr: 0.8 vs. 1.5 mg/dl^b^, BUN: 11 vs. 30 mg/dl^b^	ECCO2R: 49.2 ml/min^b^, PaCO_2_: 47.8 vs. 66.2 mm Hg^b^	Blood pH: 7.41 vs. 7.26^b^	N/A	N/D	ICU: 45%	^35^

Abbreviations: BUN, blood urea nitrogen; CI, cardiac index; CK, creatinine kinase; CL_T_, total clearance; CPI, cardiac power index; CPP, cerebral perfusion pressure; DP, driving pressure; ECCO2R, extracorporeal CO_2_ removal; FiO_2_, fraction of inspired oxygen; ${\mathrm{HCO}}_{3}^{-}$, serum bicarbonate; MAP, mean arterial pressure; N/A, not applicable; N/D, not documented; N/N, nitrate/nitrite; NA, noradrenaline; NH_3_, ammonia; PaCO_2_, CO_2_ arterial pressure; SCr, serum creatinine; *t*_1/2_, half‐life; UO, urine output.

^a^ ADVOS versus control group.

^b^ After versus before ADVOS.

^c^ Matched historical cohort as control group.

^d^ According to SOFA Score prior to ADVOS treatment.

^e^ According to CLIF‐ACLF Score prior to ADVOS treatment.

### 2.1 Extra‐corporeal albumin dialysis with ADVOS

Liver support systems have been shown to reduce mortality in patients with confirmed liver failure.^9^ High‐volume plasma exchange improves outcomes in acute liver failure (ALF) but is expensive and associated with bleeding and hypotension.^18^ An alternative strategy is extra‐corporeal albumin dialysis (ECAD), in which the dialysis circuit is supplemented with human albumin, which has a role in expanding the circulatory volume but also serves as a scavenger to remove circulating protein‐bound toxins, such as cytokines, bile acids, and endotoxins.

The Molecular Adsorbent Recirculating System (MARS) was initially introduced in 1993 and is perhaps the most clinically utilized ECAD device.^19^ Its beneficial effects in the management of hepatic encephalopathy, pruritus, and hemodynamic instability are well‐established,^20^ although limited dialysis and detoxification capabilities have been reported as a drawback.

The ADVOS system (ADVITOS, GmbH, Munich, Germany) is the first and currently, only device integrating simultaneous organ support for the liver, lungs, and kidney alongside blood pH management. This advanced hemodialysis system incorporates an extra‐corporeal blood circuit, a dialysis circuit, and a dialysate regeneration circuit (ADVOS multi‐circuit) to allow the removal of hepatic toxins and water‐soluble and protein‐bound nephrotoxins, and additionally facilitates acid–base regulation by the direct removal of blood acid and extra‐corporeal elimination of CO_2_. The CE‐marked and patented technology is marketed by the company ADVITOS, itself founded in 2005 by nephrologist Bernhard Kreymann and mechanical engineer Catherine Schreiber. Presently, the adoption of ADVOS therapy has been limited to German healthcare settings, although expansion throughout continental Europe is anticipated.

### 2.2 Mechanism of action

The ADVOS multi‐system is indicated for patients with acute, chronic, and acute‐on‐chronic liver failure or renal failure, especially to remove water‐soluble and protein‐bound toxic substances, normalize, or improve the composition of blood in case of electrolyte or acid–base disturbances, and remove fluids in case of fluid overload, as stated in the manufacturer’s instructions. This includes fluid‐based CO_2_ removal, by means of H^+^ and ${\mathrm{HCO}}_{3}^{-}$ balancing.^21^

**FIGURE 1:**
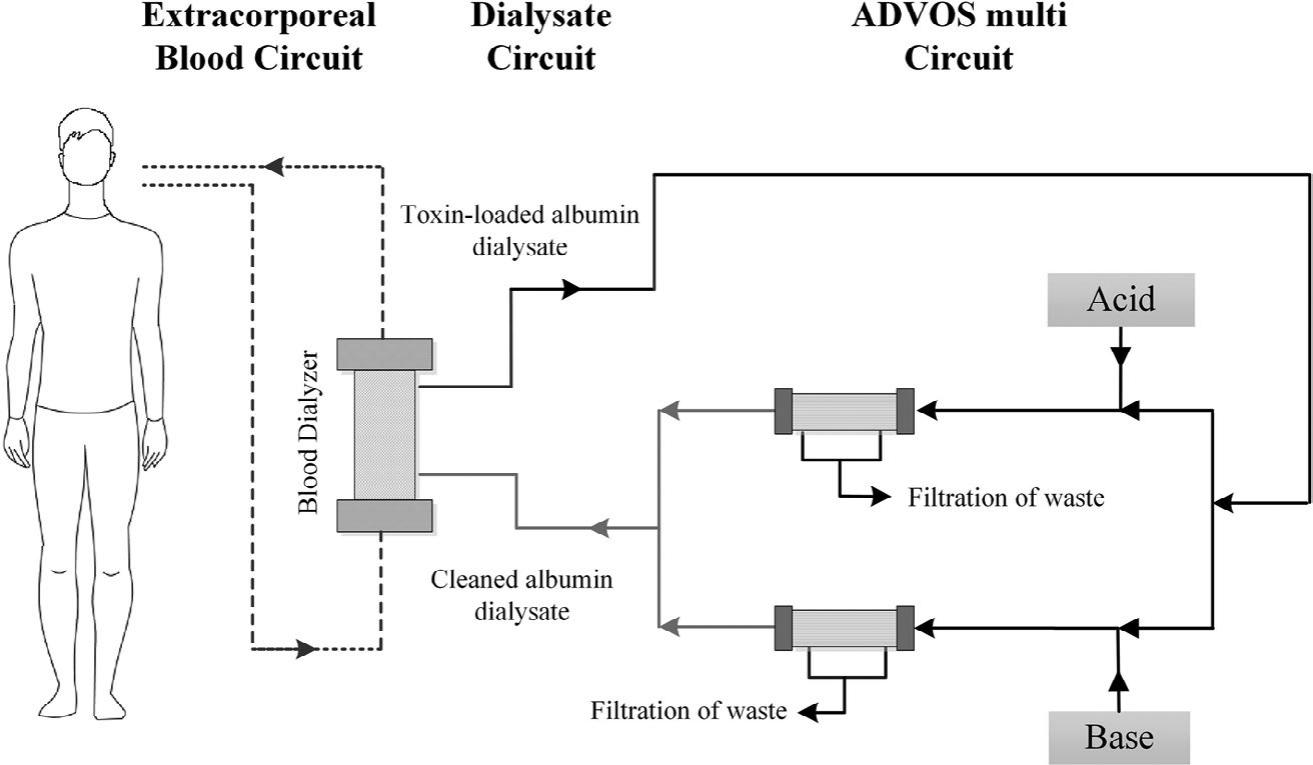
Schematic of the advanced organ support (ADVOS) system

The system comprises three communicating circuits (Figure 1). In the first extra‐corporeal blood circuit, blood is passed over two 1.9 m^2^ high flux filters and recirculated back to the patient via a double‐lumen dialysis catheter (e.g., 13 Fr). Blood flows can be adjusted between 100 and 500 ml/min. The second dialysis circuit is perfused at 800 ml/min by dialysate with 200 ml of a 20% albumin solution running in parallel to the first circuit and separated from it by the semi‐permeable dialyzer membrane. This arrangement enables the effective removal of protein‐bound toxins. In contrast to a conventional single‐pass albumin dialysis, the toxin‐loaded dialysate is not discarded but it instead enters the third circuit of the ADVOS system. Here, the dialysate is separated into two branches, where the addition of acidic or alkalic concentrates mixed with osmosis water at 160–320 ml/min flows, and temperature modifications, alter the binding site availability of albumin and induce conformational change in its tertiary structure, respectively. This facilitates the release of cationic and anionic albumin‐bound toxins, which are removed by convection through two 1.3 m^2^ dialyzers. The acid and alkali branches of the third circuit finally converge so that unloaded albumin dialysate is reintroduced into the affluent limb of the second circuit at a tailored pH. Flow rates and acid/alkali mixing ratios may be customized to generate a dialysate with pH 7.2–10.0. In addition, the bicarbonate level of the dialysate can be adjusted through different alkalic concentrates. Altogether, this permits the effective correction of metabolic or respiratory acidosis and fluid‐based extra‐corporeal CO_2_ removal. All added and replaced fluids are continuously balanced on top of the ultrafiltration rate set. The permeate (osmosis water) is provided in a movable container under the machine, where the used filtrate accumulates in a separate bag. This makes ADVOS multi a more flexible device as it is not fixed to a specific water source.

Thus, several special features of the ADVOS system exist to enhance its performance and detoxification capabilities. Firstly, the utilization of albumin‐rich dialysis solution rather than conventional dialysis fluid facilitates protein‐bound toxin clearance. The continued physical and biochemical regeneration of the albumin dialysate in the third ADVOS circuit means that it can be recycled for further toxin binding. Customisable temperature and pH modulation of the dialysate at this stage together with a variety of alkalic concentrates with 0, 10, or 20 mmol/L of bicarbonate represents an additional therapeutic opportunity in critically ill patients if deemed necessary. ADVOS treatments may be performed for up to 24 h with just 200 ml of 20% albumin solution and at low blood flow rates, thus mitigating the bleeding and hemolysis risks otherwise frequently encountered at higher flow rates. Finally, anticoagulation for dialysis is usually employed based on clinical judgment, for example with unfractionated heparin or regional citrate. A typical anticoagulation protocol for ADVOS treatments can be found in the supplementary information of Ref. [22].

### 2.3 Animal studies with ADVOS

Al‐Chalabi and colleagues evaluated the safety and efficacy of Hepa Wash (Hepa Wash GmbH, München, Germany), a novel liver support system based on albumin dialysis and precursor of ADVOS, in a highly standardized porcine model of ALF.^23^ Following the establishment of experimental ALF,^24^ six pigs were assigned for treatment with Hepa Wash, and five were allocated as a control group. The authors observed that cerebral perfusion pressures after 8 h of Hepa Wash treatment (23 ± 2 vs. 10 ± 3 mm Hg, *p* = 0.006) and mean arterial pressures (37 ± 1 vs. 24 ± 2 mm Hg, *p* = 0.006) were less diminished following the induction of ALF in animals receiving Hepa Wash treatment compared to those in the control group. Hepa Wash was furthermore associated with a beneficial effect on cardiac index at 12 h (4.94 ± 0.33 vs. 3.36 ± 0.25 ml/min/m^2^, *p* = 0.006) and renal function, with greater urine production (1850 ± 570 ml vs. 420 ± 180 ml) noted in the Hepa Wash treatment group. The elimination of both water‐soluble (creatinine, 1.3 ± 0.2 vs. 3.2 ± 0.3 mg/dl, *p* = 0.01; ammonia, 562 ± 124 vs. 1382 ± 92 μg/dl, *p* = 0.006) and protein‐bound toxins (nitrate/nitrite levels, 5.54 ± 1.57 vs. 49.82 ± 13.27 μmol/L) at 12 h was also enhanced by Hepa Wash. Survival was significantly increased in the Hepa Wash group (*p* = 0.03). In this small sample, no adverse events attributed to Hepa Wash were identified. The authors conclude that Hepa Wash treatment can be performed safely and improves biochemical parameters, organ function, and survival in their large animal model of ALF.

Al‐Chalabi and colleagues subsequently assessed the ADVOS system in a randomized study involving five pigs allocated to treatment with ADVOS, and five pigs forming a control group, following induction of cholestatic liver injury with superimposed endotoxin administration in simulation of multi‐organ dysfunction.^25^ All pigs receiving ADVOS treatment survived the 10‐h observation period after endotoxemia. At 6 h, animals in the ADVOS group had stable and significantly higher mean arterial pressure, cardiac index (6.7 vs. 4.9 ml/min/m^2^), and cardiac power index (0.83 vs. 0.41 W/m^2^) compared to the control group, a significantly lower fraction of inspired oxygen required to maintain adequate oxygenation (49% vs. 82%), higher stable cerebral perfusion pressures, and reduced levels of liver (bilirubin, 2.3 vs. 5.5 mg/dl; ammonia, 194 vs. 681 μg/dl; lactate, 4.2 vs. 8.3 mmol/L) and renal (blood urea nitrogen [BUN], 6 vs. 17 mg/dl) markers. Urine output, however, was not significantly different between the ADVOS and control groups. There were no adverse events related to ADVOS. The authors concluded that ADVOS is a safe and effective treatment modality, resulting in enhanced survival and major organ function, in a large animal model of sepsis with multi‐organ dysfunction.

### 2.4 Proof of concept for fluid‐based CO_2_ removal

Utilizing an ex‐vivo porcine blood model of hypercapnia or lactic acidosis with continuous CO_2_ or lactic acid infusion, respectively, Perez Ruiz de Garibay and colleagues showed that the ADVOS system could extract 61 ml/min of CO_2_ while maintaining physiological pCO_2_ and ${\mathrm{HCO}}_{3}^{-}$ ranges, or up to 142 ml/min of CO_2_ in hypercapnic conditions with pCO_2_ greatly increased at 117 mm Hg during low blood flow.^21^ In simulated metabolic acidosis, ADVOS could compensate for an acid load of up to 3 mmol/min and normalize blood pH and ${\mathrm{HCO}}_{3}^{-}$ levels within 1 h, whereas this was not possible with either continuous venovenous hemofiltration or continuous venovenous hemodialysis (CVVHD).^21^

This paper described for the first time the hypothesized mechanism of action for acid–base balance correction and fluid‐based extracorporeal CO_2_ removal with ADVOS. The authors propose a concentration gradient for H^+^, facilitating acid transfer from blood to the alkaline dialysate (pH 9.0). This may serve to correct metabolic acidosis. Moreover, when a simultaneous gradient for ${\mathrm{HCO}}_{3}^{-}$ is established using a dialysate with less bicarbonate, fluid‐based CO_2_ removal can be instituted.

### 2.5 Human studies with ADVOS

The feasibility, efficacy, and safety of ADVOS were investigated for the first time in humans in a clinical study by Huber and colleagues.^26^ Fourteen patients with either acute‐on‐chronic or secondary liver failure underwent a total of 239 ADVOS treatments, with one patient undergoing over 100 treatment cycles. Results observed in preceding animal studies were echoed in this first‐in‐man study. After just a single ADVOS session, serum bilirubin decreased significantly by 32% (17.7 ± 10.5 vs. 26.0 ± 15.4 mg/dl, *p* = 0.001), serum creatinine was significantly lowered by a mean of 27% (1.6 ± 0.7 vs. 2.2 ± 0.8 mg/dl, *p* = 0.005) and BUN by a significant 37% (31.1 ± 20.29 vs. 49.4 ± 23.3 mg/dl, *p* = 0.003). All procedures were completed without interruption and well‐tolerated without hemodynamic compromise or other side effects. This small observational study demonstrated that ADVOS therapy is feasible in human subjects, in whom it efficiently eliminates water‐ and protein‐bound toxins associated with liver failure.

In extension, Fuhrmann and colleagues analyzed outcomes with ADVOS for the first time in the treatment of multi‐organ failure in 34 critically ill patients in a single‐institution setting.^22^ Treatment with ADVOS was associated with significant reductions in bilirubin (−17.0%; interquartile range [IQR]: −27.8%, 0.0%), serum creatinine (−7.1%; IQR: −26.7%, +6.7%), BUN (−17.6%; IQR: −44.0%, 0.0%), and ammonia (−16.4%; IQR: −36.4%, +8.5%) levels. Significant improvements in blood pH, ${\mathrm{HCO}}_{3}^{-}$, and PaCO_2_ were observed after a single ADVOS treatment in six patients with severe metabolic acidosis refractory to renal replacement therapy and progressive multi‐organ failure. Normalization of blood pH was achieved in a median of 6 (IQR 3–12) h. In a subgroup of patients with acute respiratory distress syndrome (ARDS), corrections in pH and PaCO_2_ permitted reductions in driving pressures in up to 75% of treatment sessions, in addition to maximal inspiratory pressures. A trend toward reduced tidal volume and minute ventilation was also discovered. Hemodynamic improvements (mean arterial blood pressure 74 vs. 69 mm Hg, *p* > 0.05) during ADVOS therapy resulted in a reduction of norepinephrine usage in 73% of cases, and norepinephrine was discontinued in 43% following ADVOS treatment. The 28‐ and 90‐day mortality rates in this study population with advanced multi‐organ failure and median SOFA score 17 were 50% and 62%, respectively, which the authors suggest is lower than would be anticipated based on reported data.^27^ This case series demonstrated that ADVOS is a safe treatment for effectively clearing water‐soluble and protein‐bound substances in critically ill patients, while normalizing refractory acid–base disturbances.

Falkensteiner and colleagues recently compared MARS and ADVOS in 49 critically ill patients with liver failure undergoing 75 MARS and 58 ADVOS cycles.^28^ Both systems were found to provide equivalent detoxification capacity as evidenced by similar clearance rates of bilirubin (MARS: −13%, IQR: −33.6%, −5.2%; ADVOS: −15%, IQR: −25.8%, −1.7%, *p* = 0.333) and creatinine (MARS: −18%, IQR: −26.1%, −3.0%; ADVOS: −18%, IQR: −3.2%, −2.1%, *p* = 0.638). However, a greater relative reduction in urea levels was recorded with ADVOS compared to MARS (MARS: −6%, IQR: −14.1%, +7.2%; ADVOS: −21%, IQR: −37.5%, −0.2%, *p* = 0.01). There was an overall relative increase from pre‐ to post‐treatment lactate levels with MARS (+14%, IQR: −5.9%, +35.1%) compared to ADVOS (−1%, IQR: −21.2%, +3.2%). However, there was no significant difference in any laboratory or clinical parameters between treatments when adjusting for therapy duration, and mortality rates were not influenced by treatment modality. In summary, both MARS and ADVOS devices were considered to have comparable detoxification capabilities in critically ill patients with liver dysfunction.

Another retrospective study by Kaps and colleagues evaluated the efficacy of intermittent ADVOS therapy in settings external to intensive care and compared it against conventional hemodialysis in a matched cohort.^29^ Following 16 h of treatment, BUN levels were significantly reduced (median −33.7%, *p* = 0.00012), in conjunction with serum bilirubin (median −14.5%, *p* = 0.034) and creatinine (median −11.8%, *p* = 0.04) levels. When ADVOS was compared against hemodialysis, there was a greater reduction in bilirubin levels in the ADVOS group (median −2.48 vs. 1.01 mg/dl, *p* = 0.01), whilst no difference was seen for creatinine and BUN. Furthermore, the observed 28‐day mortality in the ADVOS group of 56% judged against the predicted 28‐day survival of 44% suggests that ADVOS does not have a detrimental effect on survival. The authors propose that discontinuous ADVOS treatment outside of intensive care units provided safe and effective detoxification in patients with acute‐on‐chronic liver failure.

The last report has been published by Scharf and colleagues.^30^ This single‐center, retrospective observational study investigated the influence of ADVOS and hemadsorption therapy in critically ill patients with acute liver dysfunction (ALD) and bilirubin levels higher than 10 mg/dl. Thirty‐three patients treated with hemadsorption, and 6 patients treated with ADVOS were included in the study. Hemadsorption reduced bilirubin levels in 22.5% while ADVOS achieved a reduction of 22.8%. In addition, hemadsorption significantly reduced serum alanine aminotransferase (ALT), serum aspartate aminotransferase (AST), glutamine‐glutamyl transferase (GGT) levels, and norepinephrine demand. In hospital‐mortality was 66.7% for ADVOS and 84.8% for hemadsorption. The authors concluded that the use of ADVOS and hemadsorption (integrated into high‐flux dialysis) led to a significant and comparable decrease in bilirubin in this cohort.

The successful application of ADVOS has been documented in case reports.^31^, ^32^ Progressive multiorgan failure occurring with acute kidney injury, liver failure, and hepatic encephalopathy in a 21‐year‐old male patient who developed severe rhabdomyolysis while receiving risperidone therapy was treated with ADVOS in the case report by Jarczak and colleagues.^31^ They found that ADVOS reduced myoglobin and creatine kinase levels by 40.9% and 62.2%, respectively, and by 50.5% and 80.8% in combination with CytoSorb, compared to 0.8% and 11.5% using CVVHD. In another case report, this group monitored antibiotic drug levels during continuous ADVOS treatment followed by CRRT.^32^ The 75‐year‐old patient with grade 2 acute‐on‐chronic liver failure, grade 3 acute kidney injury and septic shock initiated empiric antibiotic therapy with meropenem (1 g every 8 h). ADVOS (250 ml/min blood flow) was initiated on the same day and was followed by CVVHD (100 ml/min blood flow) 2 days after. The authors concluded that during ADVOS and CVVHD, meropenem dosing of 1 g 8‐hourly provided trough levels of 4.1–9.4 mg/L. They suggested prolonged infusion rates during ADVOS to achieve 100% *f*T > 1–4× MIC (percent of time that free drug remains above 1–4 times the minimum inhibitory concentration), corresponding to meropenem trough levels of 2–8 mg/L when targeting gram‐negative bacteria including *Pseudomonas aeruginosa*. Therapeutic drug monitoring is valuable here because ADVOS will affect the elimination and therefore circulating blood levels of antimicrobials.

Finally, a multi‐center, non‐interventional registry has been established since January 2017 to collect data on the performance and safety of ADVOS when employed in adult patients aged ≥18 years requiring multi‐organ support.^33^ More specifically, information on clinical laboratory tests, vital signs, health status, liver function, and ADVOS treatment parameters are collected at hospital admission, at baseline immediately prior to the first ADVOS treatment, after the first treatment, on days 1, 3, 7, 28, and 90 after the first treatment. At the time of the first interim report, the registry had incorporated 118 patients enrolled across four centers in Germany, with median of three failing organs, median SOFA score of 14 (IQR: 11,16), and predicted mortality 80%, who underwent 429 ADVOS treatment sessions. This 2‐year interim analysis has demonstrated promising outcomes.^33^ Significant reductions in serum creatinine (1.5 vs. 1.2 mg/dl, *p* < 0.001), BUN (24 vs. 17 mg/dl, *p* < 0.001), and bilirubin (6.9 vs. 6.5 mg/dl, *p* < 0.001) were recorded following the first ADVOS treatment session. ADVOS facilitated the normalization of blood pH, bicarbonate, and base excess in patients with acid–base derangement without altering pCO_2_. The registry reported a 28‐ and 90‐day mortality of 60.3% and 64.6%, respectively, after the first ADVOS treatment session. Applying a multivariable logistic regression model, a higher initial SOFA score immediately before the first ADVOS session was correlated with greater mortality risk, suggesting that a more favorable prognosis can be attained when ADVOS is not instituted as a last line treatment option. Regarding safety, non‐serious clotting of the device occurred as adverse events in only 13 cases, and significant reductions in platelet count were perceived as a generic phenomenon arising with dialysis treatments. The authors deemed ADVOS as safe based on the 6800 h of treatment included in the registry data.

### 2.6 ADVOS applications during COVID‐19

In the current era of the COVID‐19 pandemic, a minority of infected patients will progress to fulminant respiratory failure featuring ARDS. Extra‐corporeal CO_2_ removal with ADVOS has recently been appraised first in a case report and then in a case series with nine COVID‐19 patients. In the case report by Huber and colleagues, an 80‐year‐old male patient with COVID‐19 infection was treated with ADVOS for progressive ARDS and multi‐organ failure characterized by renal dysfunction, septic shock, hepatic injury, and mixed acidosis.^34^ ADVOS proved effective at CO_2_ removal with mean estimated CO_2_ elimination rate of 48 ± 23 ml/min, and significant differences between mean arterial and post‐dialyser pCO_2_ (69 ± 14 vs. 27 ± 12 mm Hg, *p* < 0.001). ADVOS‐dependent pH regulation permitted weaning of vasopressor requirements, and hemodynamic parameters were sufficiently improved after 95 h of continuous ADVOS therapy for a reduction in the noradrenaline infusion rate to 0.04 μg/kg/h compared to 0.35 μg/kg/h initially. Although the patient succumbed following a cardiac arrest, this case nevertheless supports the feasibility and efficacy of ADVOS in CO_2_ removal and acidosis correction.

Nine patients with a median age of 60 (IQR: 53–77) years with COVID‐19 infection‐related severe respiratory insufficiency and CO_2_ retention undergoing 137 ADVOS treatment sessions were documented in a later case series by Allescher and colleagues.^35^ ADVOS enabled an accelerated rectification of acid–base disturbance, with significant improvement in blood pH (7.26 vs. 7.41, *p* = 0.003) after just one ADVOS treatment. Similarly, a median continuous CO_2_ removal of 49.2 ml/min (IQR: 26.9–72.3 ml/min) and a consequent PaCO_2_ reduction (66.2 vs. 47.8 mm Hg, *p* = 0.017) was observed, with the rate of CO_2_ extraction correlating positively with blood flow, PaCO_2_, and ${\mathrm{HCO}}_{3}^{-}$ levels. Thus, even severe respiratory acidosis could be efficiently corrected without significant compensatory modification of ventilatory parameters. ADVOS proved effective in the removal of water‐soluble substances, as denoted by significant reductions in creatinine (1.5 vs. 0.8 mg/dl, *p* = 0.01) and BUN (30 vs. 11 mg/dl, *p* = 0.003). Mortality was reported at 55% in this group. The authors propose ADVOS as a feasible technique for CO_2_ removal in patients with COVID‐19 ARDS and multi‐organ failure.

## 3 DISCUSSION

ADVOS therapy was first conceived to improve therapeutic alternatives for the removal of water‐soluble and protein‐bound metabolites and toxins. This is reflected in preliminary animal studies and the first clinical studies demonstrating the proof of concept,^23^, ^25^, ^26^ which fulfilled claims regarding bilirubin and creatinine elimination. Indeed, recent observational analyses have addressed the capability of ADVOS in patients with liver failure in need of dialysis, who need clearance of water‐soluble and protein‐bound toxins.^28^, ^30^ On the one hand, Falkensteiner and colleagues retrospectively analyzed ADVOS against MARS and found a comparable efficacy regarding bilirubin or creatinine removal. In fact, this could be expected as both implement albumin dialysis. However, patients with acidosis were not included, so any influence of ADVOS on this could not be detected. On the other hand, Scharf and colleagues analyzed outcomes in 6 versus 33 patients undergoing ADVOS and CytoSorb therapy, respectively, for acute liver dysfunction in critical illness.^30^ With such a small patient population in the case of ADVOS, any possibility of finding significant differences between groups becomes difficult. Both ADVOS and CytoSorb however achieved a 22% reduction of bilirubin. However, this did not have a great impact on survival as in‐hospital mortality was 66.7% using ADVOS and 84.8% using CytoSorb. Again, due to the small number of patients involved, it is challenging to draw any meaningful conclusion. In contrast to these reports, Kaps and colleagues carried out a comparison of 25 matched patients with liver failure and need for renal replacement therapy treated either with ADVOS or intermittent dialysis got up to 8 h from a historical cohort outside the ICU.^29^ Here, ADVOS showed an improvement regarding protein‐bound toxin removal. What is more, in comparison to the control group, a trend for improved survival was shown, especially in long‐term data. Nevertheless, the differences were not significant (*p* = 0.08), probably due to limited numbers of patients included.

In all these studies, no references to CO_2_ removal or acid–base balance correction can be found. The therapeutic possibilities of modulating dialysate pH with ADVOS may only have been used to regenerate albumin, but were not applied as a therapeutic option. The development of the technology to fully control the dialysate pH, turned the perspective of the liver support therapy into the multiorgan approach, including lung support through fluid‐based CO_2_ removal and acid–base balance correction. These features were first described by Perez and colleagues in 2019.^21^ The authors based their explanation in a simple concentration gradient for H^+^ and, optionally, for ${\mathrm{HCO}}_{3}^{-}$. In this way, pH might be corrected and, if needed, ${\mathrm{HCO}}_{3}^{-}$ would be removed during hypercapnic acidosis, or augmented in case of metabolic acidosis, for example.

This customizable feature may represent the most differentiating factor of ADVOS as a multi‐organ support therapy. First, ADVOS is able to correct metabolic acidosis without an increased load of CO_2_ as occurs during a conventional bicarbonate infusion, therefore avoiding a potential increase in ventilation parameters. Second, respiratory acidosis can be corrected at low blood flows without the need for an oxygenator and a blood‐gas contact. This allows the use of conventional dialysis catheters (13 Fr) with regional anticoagulation. The data from Fuhrmann in 2020 showed this evolution.^22^ Fuhrmann et al analyzed data from 34 patients and performed subgroup analyses for patients with ARDS and with severe metabolic acidosis. In both cases, ADVOS managed to improve blood pH within 6 h. In ARDS patients, a reduction in PaCO_2_ and an improvement in driving pressure were demonstrated for the first time. In patients with metabolic acidosis, the correction of base excess and serum bicarbonate levels was also proven.

Moreover, the COVID pandemic has accelerated the obtention of results regarding extracorporeal CO_2_ removal and lung support. Recent case reports have presented the possibilities of ADVOS in the management of COVID‐related respiratory failure.^34^, ^35^ In the case series by Allescher and colleagues, a median CO_2_ removal of 49 ml/min was reported using ADVOS with elevated dialysate pH 8.5–9.0, blood flows around 300 ml/min, and regional citrate anticoagulation. This resulted in normalization of physiological blood pH levels and a significant reduction of PaCO_2_. This improvement was accompanied by a concomitant removal and significant reduction of creatinine or urea, indicating that ADVOS might be beneficial in patients with COVID‐19 multiple organ failure with superimposed CO_2_ retention.

The largest study to date refers to the 2‐year interim analysis of the registry of patients undergoing extracorporeal organ support with ADVOS.^33^ Data were obtained from a critically ill heterogenous patient population with multiple organ failure and indication for dialysis with ADVOS. Despite the limitations of the registry with a relatively small patient population and lack of a control group, it nevertheless represents a useful source of real‐world evidence, where ADVOS is shown as an efficient and safe therapy. As the authors point out, these results might not be generalizable and subgroup analysis would be intriguing, especially in patients with acid–base disorders, which were reported in >50% of the patients included.

## 4 CHALLENGES AND FUTURE DIRECTIONS

Unfortunately, almost all the current data on ADVOS therapy originates from small, uncontrolled clinical trials, and observational studies. Although the data are promising, larger randomized studies are needed to discern the full extent of possibilities of the ADVOS device. Future work should focus on concretizing the surrogate markers where ADVOS could be beneficial, especially in the target groups of patients for the therapy. To date, ADVOS has been mainly applied in patients with liver failure needing dialysis in German university hospitals. The reimbursement of healthcare institutions utilizing ADVOS as a liver support system could enhance its adoption across more widespread geographical locations. According to a study by Weismann and colleagues, annual incidences of extracorporeal liver support use remained stable between 0.39/100 000 in 2007 and 0.47/100 000 ELS in 2015.^36^ The authors indicated that in Germany, these therapies are not restricted to specific tertiary care or transplant centers but may be performed by any ICU and these incidences of usage might not be transferable to other high‐income countries due to limited access to extracorporeal therapy or limited reimbursement by the responsible health system providers. Therefore, the application of extracorporeal CO_2_ removal in the absence of liver support requirements remains challenging. Emerging data from COVID‐19 settings, and small clinical trials that are currently active (ADVOVENT DRKS00015874) or in the late planning phase to utilize ADVOS for ultraprotective ventilation, prophylactic acidosis correction and/or hemodynamic recovery among others, could serve as a starting point to obtain valuable information for the design of larger studies. The conduction of an adequately designed randomized trial should be the ultimate aim to fully determine the possibilities of ADVOS therapy, and validate the predominantly encouraging findings reported in almost all of the published literature thus far, if it is to gain generalized acceptance within the worldwide intensive care community.

## 5 CONCLUSIONS

The available data, although largely based on relatively small and non‐randomized observational studies, is supportive of ADVOS therapy as a powerful adjunct in the intensivist’s armamentarium for the management of complex multi‐organ failure. ADVOS demonstrates efficient detoxification characteristics, enabling the elimination of water‐soluble and protein‐bound toxins and metabolites, at least to at the same level as the state‐of‐the‐art therapies, such as RRT or MARS. On top of this, the possibility to correct metabolic and respiratory acidosis through the fluid‐based direct removal of acid and CO_2_ represents an important clinical advantage. ADVOS permits the integration of multiple organ support systems into a single device. For patients, this translates into a less invasive supportive strategy that is well‐tolerated with a minimized side effect profile, achieves significant improvements in hemodynamic and biochemical parameters, and trends toward reduced mortality rates compared to those expected in such critically ill populations. Larger series and prospective trials are now required to validate the encouraging findings from the smaller studies analyzed here, and additionally to select the most adequate surrogate markers and target populations for ADVOS.

## CONFLICT OF INTEREST

The authors declare that they have no conflicts of interest with the contents of this article.

## AUTHOR CONTRIBUTIONS

Metesh Acharya: data collection, data analysis/interpretation, drafting article, critical revision of article, approval of article. Rafal Berger: critical revision of article, approval of article. Aron‐Frederik Popov: concept/design, critical revision of article, approval of article

## Supporting information


Table S2
