# Supplementary material for: The role of the ADVanced Organ Support (ADVOS) system in critically ill patients with multiple organ failure
Source: Artif Organs. 2022 Feb 6;46(5):735–46. doi: 10.1111/aor.14188 (PMC9306712; doi:10.1111/aor.14188)
Supplement: Supplementary file 1 — Table S2 [file AOR-46-735-s001.docx]

**Supplementary Table 2:** List of research performed with the ADVOS therapy. Note: ADVOS treatment and Hepa Wash treatment refer to the same therapy.

| **Investigator** | **Year** | **Journal** | **Type of research and publication** | **Title** | **Ref** |
| --- | --- | --- | --- | --- | --- |
| Al-Chalabi A | 2010 | Critical Care | Animal Study  Abstract / Poster | Survival improvement in pigs with liver failure and superimposed sepsis by a new liver support system (Hepa Wash^®^) | 1 |
| Al-Chalabi A | 2010 | Journal of Hepatology | Animal Study  Abstract / Poster | Improvement of survival in a swine model of acute liver failure by a new liver support system (Hepa Wash^®^) | 2 |
| Al-Chalabi A | 2013 | BMC Gastroenterology | Animal Study  Article | Evaluation of the Hepa Wash^®^ treatment in pigs with acute liver failure | 3 |
| Henschel B | 2015 | Critical Care | Human Study  Abstract / Poster | First clinical experience with a new type of albumin dialysis: the HepaWash^®^ system | 4 |
| Tariparast P | 2016 | DIVI 2016 – Abstract book | Human Study  Abstract / Poster | Anwendung eines neuartigen Hämodialyseverfahrens bei einem septischen Patienten mit hyperkapnischem Lungenversagen | 5 |
| Huber W | 2016 | DIVI 2016 – Abstract book | Human Study  Abstract / Poster | Erste Erfahrungen mit dem ADVOS^®^-Verfahren (Advanced Organ Support) bei Patienten mit alkoholischer Steatohepatitis (ASH) | 6 |
| Jarczak D | 2016 | DIVI 2016 – Abstract book | Human Study  Abstract / Poster | Erfolgreiche Therapie einer schwerwiegenden Rhabdomyolyse nach Einnahme von Risperidon durch den Einsatz verschiedener Dialyse- und Adsorptionsverfahren (ADVOS^®^, CVVHD, CytoSorb^®^) | 7 |
| Fuhrmann V | 2016 | DIVI 2016 – Abstract book | Human Study  Abstract / Poster | Erste Erfahrungen mit einem neuen erweiterten Dialyseverfahren bei kritisch kranken Patienten mit Multiorganversagen | 8 |
| Sollinger D | 2016 | DIVI 2016 – Abstract book | Human Study  Abstract / Poster | Das Advanced Organ Support (ADVOS) Verfahren in der Therapie des Multiorganversagens | 9 |
| Perez A | 2017 | Critical Care | Ex vivo Model  Abstract / Poster | Combined removal of 4.5 mol/day of protons and protein bound and water soluble substances in an ex vivo model for metabolic acidosis using an Advanced Organ Support (ADVOS) system based on albumin dialysis | 10 |
| Perez A | 2017 | Critical Care | Ex vivo Model  Abstract / Poster | Advanced Organ Support (ADVOS) based on albumin dialysis, a new method for CO2 removal and pH stabilization | 11 |
| Huber W | 2017 | BMC Gastroenterology | Human Study  Article | First clinical experience in 14 patients treated with ADVOS: a study on feasibility, safety and efficacy of a new type of albumin dialysis | 12 |
| Al-Chalabi A | 2017 | Intensive Care Medicine Experimental | Animal Study  Article | Evaluation of an ADVanced Organ Support (ADVOS) system in a two-hit porcine model of liver failure plus endotoxemia | 13 |
| Fuhrmann V | 2018 | Critical Care | Human Study  Abstract / Poster | ADVOS reduces liver and kidney disease markers and corrects acidosis: the Hamburg experience | 14 |
| Perez A | 2018 | Blood Purification | Ex vivo Model  Abstract / Poster | ADVOS Corrects Respiratory Acidosis Mimicking the Body’s Renal Compensation: In Vitro Demonstration | 15 |
| Perez A | 2018 | Blood Purification | Ex vivo Model  Abstract / Poster | ADVOS Reverses Metabolic Acidosis Without Elevating pCO2: Proof of Concept In Vitro | 16 |
| Perez A | 2018 | Blood Purification | Ex vivo Model  Abstract / Poster | The ADVOS Device: Albumin Dialysis Based Approach for Significant and Continuous Removal of Water Soluble and Protein Bound Toxins Even at Low Blood Flows | 17 |
| Perez A | 2018 | DIVI 2018 – Abstract book | Ex vivo Model  Abstract / Poster | ADVOS kehrt die metabolische Azidose um, ohne pCO2 zu erhöhen: Proof of Concept in vitro | 18 |
| Perez A | 2018 | DIVI 2018 – Abstract book | Ex vivo Model  Abstract / Poster | ADVOS ahmt die Nierenkompensation des Körpers für die Behandlung der respiratorischen Azidose nach: In-vitro-Demonstration | 19 |
| Perez A | 2018 | DIVI 2018 – Abstract book | Ex vivo Model  Abstract / Poster | Das ADVOS-System: Albumin-Dialyse-basierter Ansatz zur signifikanten und kontinuierlichen Entfernung von wasserlöslichen und proteingebundenen Toxinen auch bei geringen Blutflüssen | 20 |
| Fuhrmann V | 2018 | DIVI 2018 – Abstract book | Human Study  Abstract / Poster | Erfahrungen mit Advanced organ support (ADVOS) bei Patienten mit Multiorganversagen | 21 |
| Huber W | 2018 | Intensive Care Medicine Experimental | Human Study  Abstract / Poster | The impact of connection to the extracorporeal circuit on haemodynamics (transpulmonary thermodilution (TPTD) and pulse contour analysis (PCA)) in patients treated with the ADVOS/ HepaWash-device: the HAEMADVOS-I-study | 22 |
| Huber W | 2018 | Intensive Care Medicine Experimental | Human Study  Abstract / Poster | Haemodynamic effects of disconnection from the "advanced organ support" (ADVOS): the HAEMADVOS-II-study evaluating the "retransfusion volume challenge option” (REVOLUTION) | 23 |
| Huber W | 2019 | Critical Care | Human Study  Abstract / Poster | Feasibility of ultrafiltration in patients treated with the ADVOS/HepaWash-device under haemodynamic monitoring with transpulmonary thermodilution (TPTD) and pulse contour analysis (PCA): The HAEMADVOS-III-study | 24 |
| Jarczak D | 2019 | Artificial Organs | Human Study  Article | Successful therapy of massive risperidone-induced rhabdomyolysis using different dialysis and adsorber devices: A case report | 25 |
| Perez A | 2019 | Intensive Care Medicine Experimental | Ex vivo Model  Abstract / Poster | ADVOS removes CO2 and corrects acidosis: Mechanism of action following a H+ and HCO3- gradient (physiological approach) or pCO2 and SID variations (quantitative approach) | 26 |
| Huber W | 2019 | Intensive Care Medicine Experimental | Human Study  Abstract / Poster | 24h-treatment for high-volume ultrafiltration and/or CO2-elimination with the ADVOS/HepaWash-device (HAEMADVOS IV) | 27 |
| Perez A | 2019 | Intensive Care Medicine Experimental | Ex vivo Model  Article | Respiratory and metabolic acidosis correction with the ADVanced Organ Support system | 28 |
| Faltlhauser A | 2019 | DIVI 2019 – Abstract book | Human Study  Abstract / Poster | ADVOS - Advanced Organ Support - Evaluierung eines neuartigen Extrakorporalverfahrens zum schnellen natriumbilanzneutralen Ausgleich metabolischer Azidosen | 29 |
| Kaps L | 2019 | DIVI 2019 – Abstract book | Human Study  Abstract / Poster | Extrakorporale Leberunterstützung mit ADVanced Organ Support (ADVOS) in Patienten mit Akut-auf-chronischem Leberversagen (ACLV) und Hepatorenalem Syndrom - Erste Ergebnisse aus einer Single Center Studie | 30 |
| Fuhrmann V | 2019 | DIVI 2019 – Abstract book | Human Study  Abstract / Poster | Ein nicht-interventionelles, multizentrisches, nicht-randomisiertes Patientenregister zur Anwendungsbeobachtung bei Multiorgan-Dialyse mit ADVOS: 2-jährige Zwischenanalyse bei 118 Patienten | 31 |
| König T | 2020 | Journal für Anästhesie und Intensivbehandlung | Human Study  Article | A case report about ADVanced Organ Support (ADVOS) in hypoxic hepatitis | 32 |
| Fuhrmann V | 2020 | Critical Care | Human Study  Abstract / Poster | A non-interventional, multicenter, non-randomized patient registry for multiple organ dialysis with the advos system: 2-year interim analysis in 118 patients | 33 |
| Falkensteiner C | 2020 | Therapeutic Apheresis and Dialysis | Human Study  Article | Comparison of the albumin dialysis devices Molecular Adsorbent Recirculating System and ADVanced Organ Support in critically ill patients with liver failure ‐ a retrospective analysis | 34 |
| Fuhrmann V | 2020 | Annals of Intensive Care | Human Study  Article | Advanced organ support (ADVOS) in the critically ill: first clinical experience in patients with multiple organ failure | 35 |
| Huber W | 2020 | The International Journal of Artificial Organs | Human Study  Article | Extracorporeal multiorgan support including CO2-removal with the ADVanced Organ Support (ADVOS) system for COVID-19: A case report | 36 |
| Fuhrmann V | 2021 | Medicine (Baltimore) | Human Study  Article | Registry on extracorporeal multiple organ support with the advanced organ support (ADVOS) system: 2-year interim analysis | 37 |
| Kaps L | 2021 | PLoS One | Human Study  Article | Applicability and safety of discontinuous ADVanced Organ Support (ADVOS) in the treatment of patients with acute-on-chronic liver failure (ACLF) outside of intensive care | 38 |
| Scharf C | 2021 | Scientific Reports | Human Study  Article | Successful elimination of bilirubin in critically ill patients with acute liver dysfunction using a cytokine adsorber and albumin dialysis: a pilot study | 39 |
| König C | 2021 | The International Journal of Artificial Organs | Human Study  Article | Pharmacokinetics of meropenem during advanced organ support (ADVOS ®) and continuous renal replacement therapy | 40 |
| Allescher J | 2021 | Artificial Organs | Human Study  Article | Extracorporeal carbon dioxide Removal (ECCO 2 R) with the Advanced Organ Support (ADVOS) system in critically ill COVID-19 patients | 41 |

**Reference List for Supplementary Table 2**

1. Al-Chalabi A, Matevossian E, Preissel AK, Yan H, Geiger A, Nairz E, et al. Survival improvement in pigs with liver failure and superimposed sepsis by a new liver support system (Hepa Wash®). Crit Care 2010;14:P508. doi: 10.1186/cc8740
2. Al-Chalabi A, Matevossian E, Preissel A-K, Yang Z, Schreiber C, Schimmel P, et al. 819 IMPROVEMENT OF SURVIVAL IN A SWINE MODEL OF ACUTE LIVER FAILURE BY A NEW LIVER SUPPORT SYSTEM (HEPA WASH®). J Hepatol 2010;52:S319-S320. doi: 10.1016/S0168-8278(10)60820-3
3. Al-Chalabi A, Matevossian E, v Thaden A-K, Luppa P, Neiss A, Schuster T, et al. Evaluation of the Hepa Wash® treatment in pigs with acute liver failure. BMC Gastroenterol 2013;13:83. doi: 10.1186/1471-230X-13-83
4. Henschel B, Schmid R, Huber W. First clinical experience with a new type of albumin dialysis: The HepaWash® system. Crit Care 2015;19:P383. doi: 10.1186/cc14463
5. Tariparast P, Roedl K, Horvatits T, Drolz A, Rutter K, Jarczak D, et al. Anwendung eines neuartigen Hämodialyseverfahrens bei einem septischen Patienten mit hyperkapnischem Lungenversagen. Poster presented at: 16th Congress of the German Interdisciplinary Association for Intensive Care and Emergency Medicine; 2016 Nov 30-Dec 2; Hamburg, Germany.
6. Huber W, Henschel B, Lahmer T, Braun M, Mayr U, Schmid R, et al. Erste Erfahrungen mit dem ADVOS®-Verfahren (Advanced Organ Support) bei Patienten mit alkoholischer Steatohepatitis (ASH). Poster presented at: 16th Congress of the German Interdisciplinary Association for Intensive Care and Emergency Medicine; 2016 Nov 30-Dec 2; Hamburg, Germany.
7. Jarczak D, Wehmeyer MH, Roedl K, Tariparast P, Horvatits T, Drolz A, et al. Erfolgreiche Therapie einer schwerwiegenden Rhabdomyolyse nach Einnahme von Risperidon durch den Einsatz verschiedener Dialyse- und Adsorptionsverfahren (ADVOS®, CVVHD, CytoSorb®). Poster presented at: 16th Congress of the German Interdisciplinary Association for Intensive Care and Emergency Medicine; 2016 Nov 30-Dec 2; Hamburg, Germany.
8. Fuhrmann V, Drolz A, Horvatits T, Roedl K, Horvatits K, Benten D, et al. Erste Erfahrungen mit einem neuen erweiterten Dialyseverfahren bei kritisch kranken Patienten mit Multiorganversagen. Poster presented at: 16th Congress of the German Interdisciplinary Association for Intensive Care and Emergency Medicine; 2016 Nov 30-Dec 2; Hamburg, Germany.
9. Sollinger D, Dörken M, Lutz J. Das Advanced Organ Support (ADVOS) Verfahren in der Therapie des Multiorganversagens. Poster presented at: 16th Congress of the German Interdisciplinary Association for Intensive Care and Emergency Medicine; 2016 Nov 30-Dec 2; Hamburg, Germany.
10. Perez Ruiz de Garibay A, Ende-Schneider B, Schreiber C, Kreymann B. P521 Combined removal of 4.5 mol/day of protons and protein bound and water soluble substances in an ex vivo model for metabolic acidosis using an Advanced Organ Support(ADVOS) system based on albumin dialysis. 37th International Symposium on Intensive Care and Emergency Medicine (part 3 of 3). Crit Care 2017;21:58. doi: 10.1186/s13054-017-1629-x
11. Perez Ruiz de Garibay A, Ende-Schneider B, Schreiber C, Kreymann B. Advanced Organ Support (ADVOS) based on albumin dialysis, a new method for CO2 removal and pH stabilization. 37th International Symposium on Intensive Care and Emergency Medicine (part 1 of 3). Crit Care 2017;21:57. doi: 10.1186/s13054-017-1628-y
12. Al-Chalabi A, Matevossian E, von Thaden A, Schreiber C, Radermacher P, Huber W, et al. Evaluation of an ADVanced Organ Support (ADVOS) system in a two-hit porcine model of liver failure plus endotoxemia. Intensive Care Med Exp;2017:5:31. doi: 10.1186/s40635-017-0144-3
13. Huber W, Henschel B, Schmid R, Al-Chalabi A. First clinical experience in 14 patients treated with ADVOS: a study on feasibility, safety and efficacy of a new type of albumin dialysis. BMC Gastroenterology 2017;17:32. doi: 10.1186/s12876-017-0569-x
14. Fuhrmann VH, Jarczak D, Boenisch O, Kluge S. ADVOS reduces liver and kidney disease markers and corrects acidosis: the Hamburg experience. 38th International Symposium on Intensive Care and Emergency Medicine. Crit Care 2018;22(Suppl 1):82.
15. Perez Ruiz de Garibay A, Ende-Schneider B, Kreymann B. ADVOS Corrects Respiratory Acidosis Mimicking the Body’s Renal Compensation: In Vitro Demonstration: 36th Vicenza Course on AKI & CRRT. Blood Purif 2018;46:163-186.
16. Perez Ruiz de Garibay A, Ende-Schneider B, Kreymann B. ADVOS Reverses Metabolic Acidosis Without Elevating pCO2: Proof of Concept In Vitro: 36th Vicenza Course on AKI & CRRT. Blood Purif 2018;46:163-186.
17. Perez Ruiz de Garibay A, Honigschnabel J, Kreymann B. The ADVOS Device: Albumin Dialysis Based Approach for Significant and Continuous Removal of Water Soluble and Protein Bound Toxins Even at Low Blood Flows: 36th Vicenza Course on AKI & CRRT. Blood Purif 2018;46:163-186.
18. Perez Ruiz de Garibay A, Ende-Schneider B, Kreymann B. ADVOS kehrt die metabolische Azidose um, ohne pCO2 zu erhöhen: Proof of Concept in vitro. Poster presented at: 18th Congress of the German Interdisciplinary Association for Intensive Care and Emergency Medicine; 2018 Dec 5-Dec 7; Leipzig, Germany.
19. Perez Ruiz de Garibay A, Honigschnabel J, Kreymann B. ADVOS ahmt die Nierenkompensation des Körpers für die Behandlung der respiratorischen Azidose nach: In-vitro-Demonstration. Poster presented at: 18th Congress of the German Interdisciplinary Association for Intensive Care and Emergency Medicine; 2018 Dec 5-Dec 7; Leipzig, Germany.
20. Perez Ruiz de Garibay A, Honigschnabel J, Kreymann B. Das ADVOS-System: Albumin-Dialyse-basierter Ansatz zur signifikanten und kontinuierlichen Entfernung von wasserlöslichen und proteingebundenen Toxinen auch bei geringen Blutflüssen. Poster presented at: 18th Congress of the German Interdisciplinary Association for Intensive Care and Emergency Medicine; 2018 Dec 5-Dec 7; Leipzig, Germany.
21. Fuhrmann VH, Weber T, Roedl K, Tariparast A, Jarczak D, Kluwe J, et al. Erfahrungen mit Advanced organ support (ADVOS) bei Patienten mit Multiorganversagen. Poster presented at: 18th Congress of the German Interdisciplinary Association for Intensive Care and Emergency Medicine; 2018 Dec 5-Dec 7; Leipzig, Germany.
22. Huber W, Leinfelder M, Lahmer T, Herner A, Mayr U, Batres-Baires G, et al. The impact of connection to the extracorporeal circuit on haemodynamics (transpulmonary thermodilution (TPTD) and pulse contour analysis (PCA)) in patients treated with the ADVOS/ HepaWash-device: the HAEMADVOS-I-study. Intensive Care Med Exp 2018;6:40. doi: 10.1186/s40635-018-0201-6
23. Huber W, Leinfelder M, Lahmer T, Herner A, Mayr U, Batres-Baires G, et al. Haemodynamic effects of disconnection from the "advanced organ support" (ADVOS): the HAEMADVOS-II-study evaluating the "retransfusion volume challenge option” (REVOLUTION). Intensive Care Med Exp 2018;6:40. doi: 10.1186/s40635-018-0201-6
24. Huber W, Leinfelder M, Lahmer T, Batres-Baires G, Rasch S, Schreiber S, et al. Feasibility of ultrafiltration in patients treated with the ADVOS/HepaWash-device under haemodynamic monitoring with transpulmonary thermodilution (TPTD) and pulse contour analysis (PCA): the HAEMADVOS-III-study: 39th International Symposium on Intensive Care and Emergency Medicine. Crit Care 2019;23(Suppl 2):72.
25. Jarczak D, Kluge S, Fuhrmann V. Successful therapy of massive risperidone-induced rhabdomyolysis using different dialysis and adsorber devices: A case report. Artif Organs 2019;43:1113-1115. doi: 10.1111/aor.13479
26. Perez Ruiz de Garibay A, Kreymann B. ADVOS removes CO2 and corrects acidosis: Mechanism of action following a H+ and HCO3- gradient (physiological approach) or pCO2 and SID variations (quantitative approach). Intensive Care Med Exp 2019;7:55. doi: 10.1186/s40635-019-0265-y
27. Huber W, Busch D, Mayr U, Herner A, Batres-Baires G, Rasch S, et al. 24h-treatment for high-volume ultrafiltration and/or CO2-elimination with the ADVOS/HepaWash-device (HAEMADVOS IV). Intensive Care Med Exp 2019;7:55. doi: 10.1186/s40635-019-0265-y
28. Perez Ruiz de Garibay A, Kellum JA, Honigschnabel J, Kreymann B. Respiratory and metabolic acidosis correction with the ADVanced Organ Support system. Intensive Care Med Exp 2019;7:56. doi: 10.1186/s40635-019-0269-7
29. Faltlhauser A, Codl D, Kullmann F. ADVOS - Advanced Organ Support - Evaluierung eines neuartigen Extrakorporalverfahrens zum schnellen natriumbilanzneutralen Ausgleich metabolischer Azidosen. Poster presented at: 19th Congress of the German Interdisciplinary Association for Intensive Care and Emergency Medicine; 2019 Dec 4-Dec 6; Hamburg, Germany.
30. Kaps L, Ahlbrand CJ, Gadban R, Kremer WM, Labenz C, Nagel M, et al. Extrakorporale Leberunterstützung mit ADVanced Organ Support (ADVOS) in Patienten mit Akut-auf-chronischem Leberversagen (ACLV) und Hepatorenalem Syndrom - Erste Ergebnisse aus einer Single Center Studie. Poster presented at: 19th Congress of the German Interdisciplinary Association for Intensive Care and Emergency Medicine; 2019 Dec 4-Dec 6; Hamburg, Germany.
31. Fuhrmann V, Perez A, Faltlhauser A, Tyczynski B, Jarczak D, Lutz J, et al. Ein nicht-interventionelles, multizentrisches, nicht randomisiertes Patientenregister bei Multiorgan-Dialyse mit ADVOS: 2-jährige Zwischenanalyse bei 118 Patienten. Poster presented at: 19th Congress of the German Interdisciplinary Association for Intensive Care and Emergency Medicine; 2019 Dec 4-Dec 6; Hamburg, Germany.
32. König T, Böhm S, Perez A, Bressem K, Spies C, Weber-Carstens S. A case report about ADVanced Organ Support (ADVOS) in hypoxic hepatitis. Journal für Anästhesie und Intensivbehandlung 2020:70-76.
33. Fuhrmann V, Perez A, Faltlhauser A, Tyczynski B, Jarczak D, Lutz J, et al. A non-interventional, multicenter, non-randomized patient registry for multiple organ dialysis with the advos system: 2-year interim analysis in 118 patients: P254. 40th International Symposium on Intensive Care and Emergency Medicine. Crit Care 2020;24(Supplement 1):87.
34. Falkensteiner C, Kortgen A, Leonhardt J, Bauer M, Sponholz C. Comparison of albumin dialysis devices molecular adsorbent recirculating system and ADVanced Organ Support system in critically ill patients with liver failure-A retrospective analysis. Ther Apher Dial 2021;25:225-236. doi: 10.1111/1744-9987.13533
35. Fuhrmann V, Weber T, Roedl K, Motaabbed J, Tariparast A, Jarczak D, et al. Advanced organ support (ADVOS) in the critically ill: first clinical experience in patients with multiple organ failure. Ann Intensive Care 2020;10:96. doi: 10.1186/s13613-020-00714-3-89712-4
36. Huber W, Lorenz G, Heilmaier M, Böttcher K, Sahm P, Middelhoff M, et al. Extracorporeal multiorgan support including CO_2_-removal with the ADVanced Organ Support (ADVOS) system for COVID-19: A case report. Int J Artif Organs 2021;44:288-294. doi: 10.1177/0391398820961781
37. Fuhrmann V, Perez Ruiz de Garibay A, Faltlhauser A, Tyczynski B, Jarczak D, Lutz J et al. Registry on extracorporeal multiple organ support with the advanced organ support (ADVOS) system: 2-year interim analysis. Medicine (Baltimore) 2021;100:e24653.
38. Kaps L, Ahlbrand CJ, Gadban R, Nagel M, Labenz C, Klimpke P, et al. Applicability and safety of discontinuous ADVanced Organ Support (ADVOS) in the treatment of patients with acute-on-chronic liver failure (ACLF) outside of intensive care. PLoS One 2021;16::e0249342. doi: 10.1371/journal.pone.0249342
39. Scharf C, Liebchen U, Paal M, Becker-Pennrich A, Irlbeck M, Zoller M, et al. Successful elimination of bilirubin in critically ill patients with acute liver dysfunction using a cytokine adsorber and albumin dialysis: A pilot study. Sci Rep 2021;11:10190. doi: 10.1038/s41598-021
40. König C, Kluge S, Fuhrmann V, Jarczak D. Pharmacokinetics of meropenem during advanced organ support (ADVOS®) and continuous renal replacement therapy. Int J Artif Organs 2021;44:783-786. doi: 10.1177/03913988211021101
41. Allescher J, Rasch S, Wiessner JR, Perez Ruiz de Garibay A, Huberle C, Hesse F, et al. Extracorporeal carbon dioxide removal with the Advanced Organ Support (ADVOS) system in critically ill COVID-19 patients. Artif Organs 2021;45:1522-1532. doi: 10.1111/aor.14044
